# Dione’s Wispy Terrain: A Cryovolcanic Story?

**DOI:** 10.3847/psj/abe7ec

**Published:** 2021-04-30

**Authors:** Cristina M. Dalle Ore, Christopher J. Long, Fiona Nichols-Fleming, Francesca Scipioni, Edgard G. Rivera Valentín, Andy J. Lopez Oquendo, Dale P. Cruikshank

**Affiliations:** 1 SETI Institute, 183 Bernardo Avenue, Ste 200, Mountain View, CA 94043, USA;; 2 NASA Ames Research Center, Moffett Field, CA 94035, USA; 3 Dartmouth College, Hanover, NH 03755, USA; 4 Department of Earth, Environmental and Planetary Sciences, Brown University, 324 Brook Street, Providence, RI 02912, USA; 5 Lunar and Planetary Institute, Universities Space Research Association, 3600 Bay Area Boulevard, Houston, TX 77058, USA; 6 Department of Astronomy and Planetary Science, Northern Arizona University, 527 S. Beaver Street, Flagstaff, AZ 86011, USA

**Keywords:** Saturnian satellites (1427), Surface ices (2117), Surface processes (2116)

## Abstract

We examine the H_2_O ice phase on the surface of Dione, one of Saturn’s icy satellites, to investigate whether it might harbor cryovolcanic activity induced by a subcrustal body of water. Several studies have searched for such a signature, as summarized in Buratti et al.; however, none has yet produced sufficient evidence to dissipate doubts. In the radiation environment characteristic of Saturn’s icy moons, the presence of crystalline H_2_O ice has been used as a marker of a high-temperature region. Because ion bombardment will, over time, drive crystalline ice toward an increasingly amorphous state, the current phase of the H_2_O ice can be used to gauge the temporal temperature evolution of the surface. We adopt a technique described by Dalle Ore et al. to map the fraction of amorphous to crystalline H_2_O ice on Dione’s surface, observed by the Cassini Visible and Infrared Mapping Spectrometer, and provide an ice exposure age. We focus on a region observed at high spatial resolution and centered on one of the faults of the Wispy Terrain, which is measured to be fully crystalline. By assuming an amorphous to crystalline ice fraction of 5% (i.e., 95% crystallinity), significantly higher than the actual measurement, we obtain an upper limit for the age of the fault of 152 Ma. This implies that the studied fault has been active in the last ~100 Ma, supporting the hypothesis that Dione might still be active or was active a very short time ago, and similarly to Enceladus, might still be harboring a body of liquid water under its crust.

## 1. Introduction

The inner icy moons of Saturn have been the focus of several studies aimed at understanding their geologic and geophysical activity by investigating their mutual interactions and their individual properties. For example, Enceladus owes its activity to the tidal interplay with Saturn and Dione (Spencer & Nimmo 2013), being locked into a 2:1 orbital resonance with Dione with respect to Saturn. Considering the nature of Enceladus’ activity through its plume outbursts, which generate the E-ring (Spahn et al. 2006), Dione is a natural target to search for possible activity on its surface as well.

Several studies have searched for signs of current activity on Dione, as summarized by Buratti et al. (2018a, 2018b). The detection of an aura surrounding the moon (Clark et al. 2008; Simon et al. 2011), the inference of a subcrustal ocean from two independent gravity analyses (Beuthe et al. 2016; Hemingway et al. 2016), and modeling of the core (Choblet et al. 2018) all suggest that Dione could be more similar to Enceladus than it appears. Unfortunately, a close look at high phase angle observations targeted to reveal the presence of plumes or an atmosphere (Buratti et al. 2018b) yielded no significant evidence.

However, bright streaks, dubbed “Wispy Terrain,” that stretch across Dione’s trailing hemisphere, indicate that the moon was at least active in geologically recent times. This system of bright tectonic faults crosscutting the underlying cratered surface (Smith et al. 1981; Plescia & Boyce 1982) and the smooth terrain on the leading hemisphere (Plescia & Boyce 1982; Plescia 1983; Kirchoff & Schenk 2015) requires a significant endogenic heat source in the recent past to explain its formation (White et al. 2017). Indeed, stratigraphic relationships between the faults and craters suggest their formation may have occurred 300–790 Ma ago (Hirata 2016). Present-day tidal dissipation, the main source of internal heat for the icy moons (Peale & Cassen 1978; Peale et al. 1979), though, is insufficient to explain the existence of these young tectonic features. Thus, constraining the timing of these features on Dione could help elucidate the tidal evolution of the Saturnian system and in turn provide clues to the age of the moons.

The morphological comparison with Enceladus’s tiger stripes led to the hypothesis that the wispy terrain could be the “fossilized” version of Enceladus’s South Pole fractures (Barr & Hammond 2015). The tiger stripes have been studied in depth and have shown a marked rise in temperature in the central crustal fissures (Spencer et al. 2006; Howett et al. 2011; Bland et al. 2015; Dhingra et al. 2017), as well as a detection of H_2_O ice in its crystalline phase (Brown et al. 2006; Newman et al. 2009). On Dione, though, there is currently no clear evidence of marked regional temperature differences as measured by the Cassini Composite Infrared Spectrometer (CIRS; Howett et al. 2018). The faults, however, have been found to expose clean H_2_O ice (Stephan et al. 2010) and are associated with a higher abundance of crystalline H_2_O ice than the surrounding dark terrain (Newman et al. 2009).

Crystalline ice is used as a marker for constraining the recent emplacement of higher-temperature material, and can therefore be used to gauge the temporal temperature evolution of the surface (Mastrapa & Brown 2006; Berdis et al. 2020). For example, during a hypervelocity impact onto an icy moon, the flash heating and cooling of the exposed melt forms crystalline water ice (Baragiola et al. 2013). When bombarded with charged particles, such crystalline ice will break into H and OH. The H atoms diffusing through the ice disrupt its structure turning it into disordered and amorphous (Mastrapa & Brown 2006). Several of the midsized Saturnian moons (MSMs), including Dione, orbit within Saturn’s magneto-sphere. Because the magnetosphere revolves much faster than these MSMs orbit Saturn, their trailing hemispheres are preferentially exposed to such ion bombardment (e.g., Paranicas et al. 2012). Thus, a hypervelocity impact onto an icy moon such as Dione will form crystalline water ice that, over time, is amorphized by ion bombardment. Indeed, Dalle Ore et al. (2015) found that some craters on Rhea showed an association between their morphology and water ice crystallinity. They showed that the young, fresh rayed crater Inktomi (12°S, 112°W) has a crater floor with high crystallinity and ejecta rays with lower values. Furthermore, their work on Obatala (1°S, 270°W), which is on the trailing hemisphere of Rhea, suggested an age of 450 Ma.

Similar to a hypervelocity impact, the formation of the extensional tectonic faults on Dione will lead to high regional temperatures that can crystallize water ice. Once endogenic activity ends, the terrain will return to normal temperatures and the exposed ice will amorphize as it is exposed to ion and meteorite bombardment. Here, we make use of the technique previously described in Dalle Ore et al. (2015) to determine the fraction of crystalline to amorphous H_2_O ice across the Wispy Terrain.^7^ In that work, the 2.0 *μ*m band was used because its shape is sensitive to phase changes in a consistent manner. These new estimates, along with irradiation fluxes at Dione, allow us to estimate the surface exposure age of the ice associated with these fractures, providing constraints on Dione’s internal evolution and related orbital evolution.

## 2. Data Preparation and Analysis

Dione was observed several times during the Cassini mission with the Visible and Infrared Mapping Spectrometer (VIMS) on board the spacecraft. VIMS consisted of two slit spectrometers, or channels, covering the visual (VIMS-V) and infrared (VIMS-IR) spectral ranges from 0.35 to 1.05 ***μ***m and 0.88–5.12 *μ*m, and an average spectral sampling of 7.3 and 16.6 nm, respectively (Brown et al. 2004; McCord et al. 2004). Each VIMS data file is three-dimensional, with two spatial dimensions (“samples,” along the horizontal direction; “lines,” along the vertical direction) and one spectral dimension, and it is commonly referred to as a hyperspectral “cube.”

Two data sets were adopted in this study. The global data set consists of a mosaic of more than 600 cubes prepared as described in the Appendix. The second data set combines 19 cubes at the highest spatial resolution (the pixel area ranging between ~0.5 and ~3.0 km^2^) focused on one of the faults, the Padua Chasmata, in the Wispy Terrain. This is located on Dione’s trailing hemisphere, approximately on the equator, at ~110°W.

H_2_O ice phase measurements have historically been based mostly on the presence of the 1.65 *μ*m band, where the absence of the band indicated that the ice was amorphous or crystalline warmer than 150 K (Grundy & Schmitt 1998). However, there are two problems in adopting the standard technique. The first is the location of a filter junction very close to the band position (Brown et al. 2004), which compromises the shape and depth of the band. The second problem is related to the sensitivity of the 1.65 *μ*m band. In fact, a fraction of only ~20% crystalline H_2_O ice (Mastrapa et al. 2008) mixed in with amorphous is sufficient for the 1.65 *μ*m band to be present, and therefore prevents precise measurements of the relative contribution of the two phases.

In order to obtain a precise measure of the fraction of amorphous to crystalline H_2_O ice on Dione, we chose to utilize the shape of the 2.0 *μ*m band instead. However, the 2.0 *μ*m band shape is also sensitive to variations in grain size, composition, and temperature, which have to be taken into account to properly determine the contribution of ice phase. The amorphous ice fraction measurements were performed applying the same technique described in Dalle Ore et al. (2015) for the craters on Rhea. We refer to the Appendix and that paper for a more detailed description of the technique as well as the background information from which it was derived.

There are three steps crucial to measuring the fraction of amorphous to crystalline H_2_O ice.

The first—*Calibration—*consisted of building grids of models to mimic the 2.0 *μ*m band shape variations while considering the grain size, composition (contamination of the ice), and temperature of the ice in different parts of the surface and covering a full range of fractions of amorphous to crystalline ice. The end member spectral variations across the surface were found by means of a clustering tool focused on the 1.5 *μ*m band region, known to be sensitive to all parameters to a similar degree.

**Figure A2.**
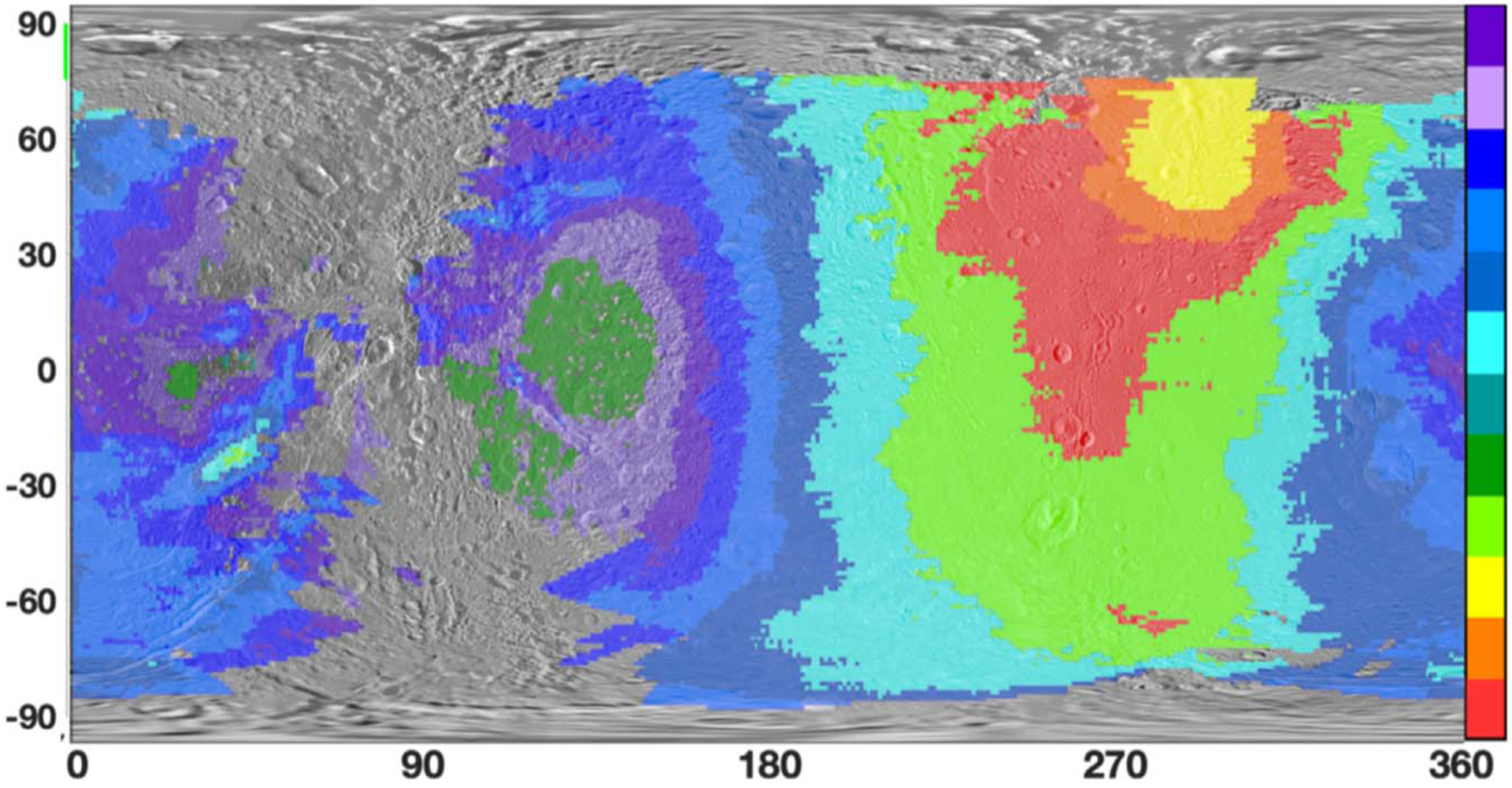
Dione cluster map showing the location of pixels that belong to the clusters identified for this data set (see legend on the right for cluster IDs from 1 to 12, starting with 1 at the bottom) overlaid upon Dione’s base map (Schenk et al. 2011).

The second step—*Delta Measurement—*consisted of measuring the change in shape in a quantitative fashion. We achieved this goal by comparing in turn all the spectra in each grid with a Gaussian fit to the 2.0 *μ*m band, taking the ratio of the spectra to the corresponding fits. We then measured the difference in normalized albedo between the two minima at 1.95 *μ*m and 2.1 *μ*m, which we named “delta.” From these measurements, we obtained the calibration curves linking delta, the asymmetry parameter, to the level of crystallinity for all the regions shown in Figure A2.

The third step—*Phase Fraction Determination—*consisted of measuring delta for all pixels in the mosaic and associating a value of the fraction of amorphous to crystalline ice making use of the appropriate calibration curve determined based on the geographical location of each pixel.

Each step is explained in detail in the Appendix.

## 3. Results

### 3.1. Low-resolution Global Amorphous H_2_O Ice Fraction Map

**Figure 1.**
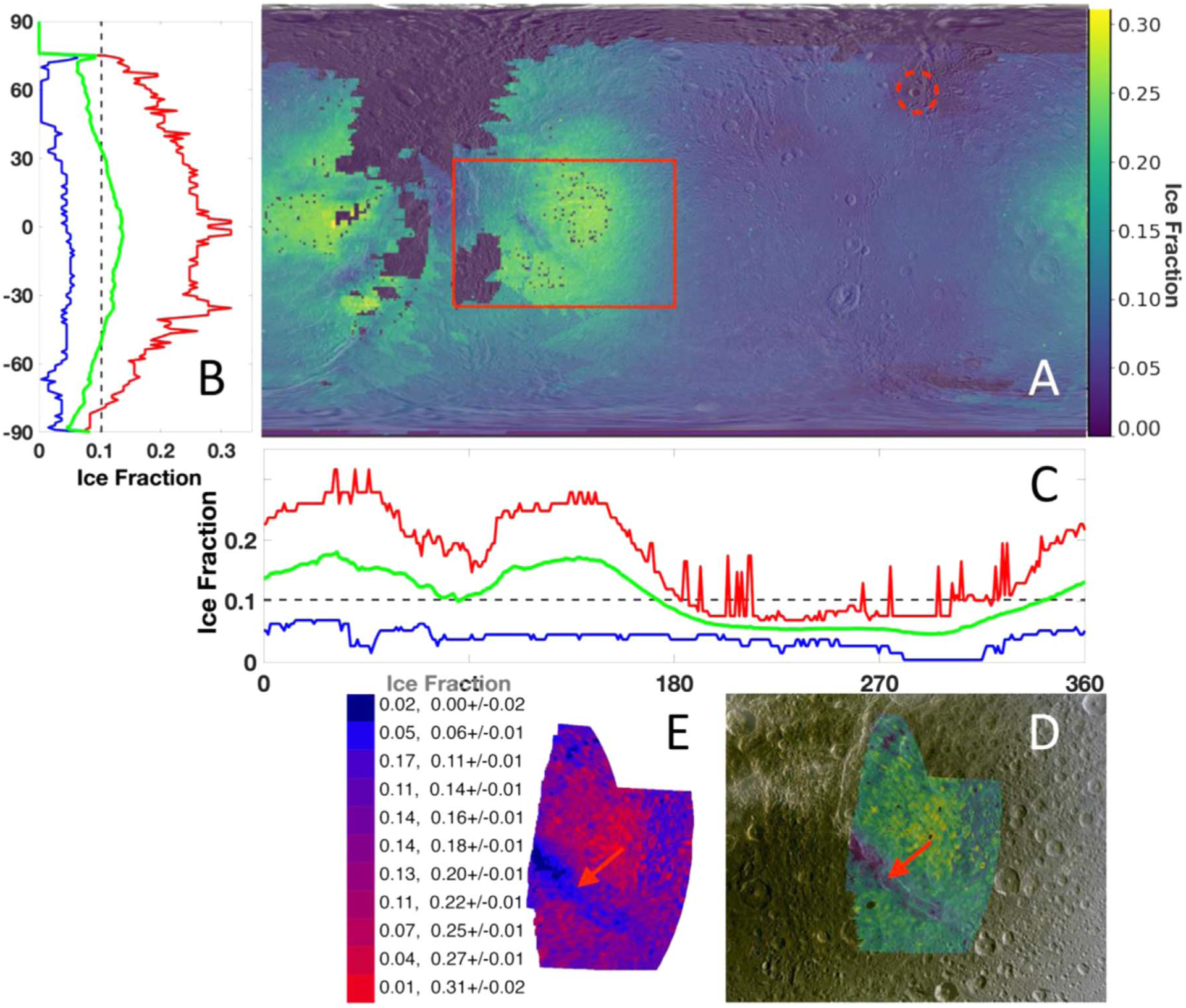
Dione global fraction of amorphous to crystalline H_2_O ice map and high-resolution subset. Panel A shows both trailing (left side) and leading hemispheres (right side). The high-resolution region and Crater Creusa are respectively marked by solid and dashed red traces. Panels B and C show the maximum (red), average (green), and minimum (blue) amorphous H_2_O ice fraction as a function of latitude and longitude respectively. Panels D and E are enlargements of the area of higher spatial resolution marked in red in panel A. The legend in panel E lists the percent spatial coverage and the corresponding amorphous fraction with its error. In panel A, the legend indicates the fraction of amorphous ice relative to crystalline.

The fraction of amorphous to crystalline H_2_O ice was measured as described in the Appendix, making use of an asymmetry parameter calibrated to the composition, grain size, and temperature of the surface region under investigation. Figure 1(A) shows the resulting map overlain on a base map of Dione (Schenk et al. 2011).

The amorphous ice distribution is unevenly spread across the surface of Dione, as displayed in panels B and C, where we plot the maximum, average, and minimum amorphous ice fraction across each latitude and longitude, respectively. The leading hemisphere (i.e., 180°–360° longitude in panel C), is predominantly crystalline (i.e., low amorphous ice fraction). This is due to the continuous infalling of E-ring particles, known to be mainly composed of crystalline H_2_O ice (Postberg et al. 2008, 2009). In this hemisphere, the rayed crater Creusa, highlighted with a red dashed line in Figure 1(A), stands out in crystallinity, i.e., low amorphous ice fraction, with a corresponding slight dip in total amorphous fraction visible in Figure 1(C). This crater is young (1–100 Ma) (Zahnle et al. 2003; Stephan et al. 2010; Scipioni et al. 2013, Hirata 2016) and therefore recently subjected to melting and slow recondensing of the H_2_O ice, conditions known to yield crystalline ice (Dalle Ore et al. 2015 and references therein).

On the trailing hemisphere (i.e., 0°–180° longitude in panel C), the H_2_O ice phase distribution is more varied and the story more complicated. Because of the position of Dione’s orbit with respect to Saturn’s magnetosphere, the trailing hemisphere is bombarded by particles that overtake the satellite and have two known effects on the surface: they darken it, due to the composition of the magnetospheric grains hitting the surface (Noll et al. 1997; Clark et al. 2008; Jaumann et al. 2009; Schenk et al. 2011; Paranicas et al. 2012), and they change the phase of the surface H_2_O ice. In fact, when crystalline ice is bombarded, its symmetric structure is disturbed, leading to the formation of disordered—or amorphous—ice.

If no other mechanism were at work on Dione, then most of the trailing hemisphere would be covered in amorphous H_2_O ice to the degree allowed by Dione’s diurnal temperatures and thermal recrystallization. As described in Loeffler et al. (2020), at temperatures of ~80 K the maximum amorphization to be expected from electrons in the 1–10 KeV range is about 30%, decreasing to less than 10% at 100K, and implying for Dione a lower amount of amorphous ice than the widespread measured ~30%. We can attribute this discrepancy to the fact that the surface is bombarded not only by electrons but also by a variety of particles with a spectrum of energies.

However, further mechanisms are at work, e.g., the flash heating and cooling during impact cratering leads to the formation of crystalline ice, which is amorphized over time by charged particle bombardment (Baragiola et al. 2013; Dalle Ore et al. 2015). On Dione’s surface, the trailing hemisphere is interrupted in several places by changes in H_2_O ice phase, some clearly corresponding to tectonic features in the Wispy Terrain region. In this region, the amount of amorphous ice decreases distinctly to just a few percent from the more common 25%–30% levels found in the neighboring parts of the hemisphere. To further analyze the nature of the ice in those areas where the ice phase varies from amorphous to crystalline, we created a mosaic of high-spatial-resolution cubes and performed the same analysis outlined in the Appendix to obtain the fraction of amorphous to crystalline ice.

### 3.2. High-resolution Wispy Terrain Amorphous H_2_O Ice Fraction Map

Figure 1(D) shows the distribution of amorphous versus crystalline H_2_O ice in and around one of the faults (Padua Chasmata) belonging to the Wispy Terrain region. The map on the right shows the high-resolution subset in comparison to the global map. The ice in the fault (identified by a red arrow in the map) is almost completely crystalline, in contrast with neighboring regions away from the tectonic features, where there is as much as ~30% amorphous ice. Figure 1(E) shows a quantitative description of the distribution of the ice phase. In the legend corresponding to this map, the percentage of coverage is reported for each fraction of amorphous to crystalline ice. It is noteworthy that pixels with amorphous ice fractions larger than 10% account for only about a quarter of the mosaic; therefore, the ice is mostly crystalline.

There is also a number of “super-crystalline” pixels (no amorphous contribution) covering an area of about 2% of the mosaic. Since the change in ice phase is driven by changes in temperature, these pixels are likely marking the position of the warmest areas in the region (Baragiola et al. 2013 and references therein). Considering the fact that the fault shown in the high-resolution mosaic spans between 35 and 50 km across, as measured on an Imaging Science Subsystem (ISS) mosaic by Schenk et al. (2011), it is clear that only high-resolution measurements can detect the very subtle changes occurring on the surface. Howett et al. (2018) report that regions of the order of 50–100 km^2^ could have gone undetected by the Cassini CIRS scans if the temperature of the ice were reported to be in the range ~105–110 K. Our best-fitting models, described in detail in the Appendix, employed optical constants between 100 and 120 K, as shown in Table A1. It becomes apparent that the limited geographic expanse of the Wispy Terrain’s faults and the relatively small enhancement in temperature corresponding to the features may conspire to hide the evidence for present-day activity on the surface of Dione.

### 3.3. How Old Are the Wispy Terrain Faults?

The mechanisms that create the crystalline ice can be diverse, but are usually due to variations (enhancement) in temperature (Ligier et al. 2016; Berdis et al. 2020, and references therein). Most frequently in the outer Solar System, they amount to impact cratering, cryovolcanic activity, or thermal relaxation (Kouchi et al. 1994; Mastrapa et al. 2013). In the case of craters, the temperature enhancement is produced by the energy transfer from the impact onto the surface. In the case of cryovolcanism, the heat is coming from an interior source and is usually localized in places that highlight the location of the crack or vent from which the material at higher temperature is outflowing, such as in the case of Pluto (Cruikshank et al. 2019; Dalle Ore et al. 2019). In this case, based on the shape of the Wispy Terrain faults, the second mechanism applies. Nonetheless, the physics behind the age determination is the same for both scenarios, because the transformation from crystalline to amorphous is due to the ion bombardment, which is characteristic of the specific environment and regulates the rate of change. Therefore, following in the steps of Dalle Ore et al. (2015), we estimated an approximate age for the Wispy Terrain region shown in the high-resolution map.

Dione’s trailing hemisphere is exposed to bombardment of charged particles that, over time, convert the crystalline H_2_O ice into its disordered amorphous phase, following an exponential behavior (Famá et al. 2010; Baragiola et al. 2013) described by:

(1) $$\Phi_{A}=\Phi_{\text{Amax}}\left(1-e^{\left(-KF\cdot t\right)}\right),$$

where Φ_*A*_ is the fraction of amorphous to crystalline ice, Φ_Amax_ is the maximum fraction of amorphous ice on the surface, *K* is a fitting parameter strongly dependent on temperature (Famá et al. 2010), *F* is the irradiation fluence, and *t* is the exposure time. H_2_O ice becomes crystalline within a few minutes above 135 K (Baragiola et al. 2013), which is a much higher temperature than expected on the surface of Dione (Howett et al. 2010). The above equation does not take into account thermal recrystallization, which at Dione’s diurnal temperatures might contribute to slow down the process of amorphization and therefore introduce an error in an age determination. On regions of Dione’s surface away from sources of heat, once amorphized, the disordered H_2_O ice phase reaches an equilibrium with the crystalline one in a time upward of that listed in Table 1 for the corresponding fraction.

Within the above described limitations and based on Equation (1), we estimated an approximate age of the Wispy Terrain based on the time it would take for irradiation to bring the fraction of amorphous ice from approximately zero (Φ_*A*_), as measured in the center of the fissure, to 5% (Φ_Amax_). The latter is a fraction that is higher than the measurement errors (shown in Figure 1(E)), which range between 1% and 2%, and is therefore significant. A few assumptions were made in our calculations. For *K*, we adopted the value corresponding to irradiation from 3 keV He^+^ particles, this choice being driven by the fact that it was the only one available at temperature around 100 K and therefore applicable to our case. The irradiation fluence, *F*, was calculated based on work by Paranicas et al. (2012). According to their measurements, the proton flux on the trailing hemisphere of Dione is expected to cover a range in energy between 30 and ~700 keV, following the distribution shown in Figure 4 of Paranicas et al. (2012). We integrated the number of protons over the relevant energy range and obtained a total flux of ~2.6 × 10^3^ protons cm^−2^ s^−1^ sr^−1^. The flux was adjusted for the number of H_2_O molecules in a volume 1 cm^2^ wide and 8 *μ*m deep. In fact, according to Mastrapa & Brown (2006), 8 *μ*m is approximately half the penetration depth for 0.8 MeV protons and is consistent with the less energetic charged particles hitting Dione’s trailing hemisphere.

Our calculations yielded an age, for a 5% fraction of H_2_O ice, of about 152 Ma, implying that the fissure will be younger than that. Table 1 shows an age “ruler” for ages corresponding to fractions increasing in steps of 5% from 0% to 30%, which is the maximum fraction of amorphous H_2_O ice recorded on Dione. In the table, all values were calculated assuming a penetration depth of 8 *μ*m and a *K* value consistent with He ion bombardment. However, because the spectrum samples different depths at different wavelengths, unprocessed material deeper than the adopted depth might be included, introducing an error in our estimate. If crystalline ice from below the 8 *μ*m irradiated layer were to influence the spectrum, then the actual age of the surface could be older than we derive. In fact, if a different value were adopted for *K* (e.g., protons) or if the depth were changed to 7 *μ*m or 9 *μ*m, the age would change to 68, 133, or 171 Ma, respectively. Furthermore, if the penetration depth was decreased, the value of *K* changed to that of protons instead of He ions, and a 1% variation in the amount of amorphous were adopted at once, the resulting age would be ~71 Ma, exposing a large degree of uncertainty in the measurement tied to the many assumptions made in the derivation—and showing which (e.g., the value of *K*) carry the most weight in the calculation.

In Table 2, we list ages for different amounts of amorphous ice shown in the high-resolution map (Figure 1(C)). Again, all values were calculated assuming a penetration depth of 8 *μ*m, and a *K* value consistent with He ion bombardment.

We should point out that, while calculating the age of the ice, we are not discussing the fact that the actual phenomenon that produced the gradient in crystallinity observed around the wispy terrain fissure is a gradient in temperature, i.e., that the ice farther out than the center of the fault is or was not warm enough to crystallize completely. Once the temperature lowers to the background one, it is only ion bombardment that can bring the ice phase back to being partially amorphous. The ages shown in Table 2 were all computed with the assumption that the ice was fully crystalline to start, which might not be accurate away from the faults. Having clarified this point, whether the cause of the phase gradient is related to different temperature or ion bombardment, the age calculation stands to demonstrate that the faults, where the fraction of amorphous H_2_O ice is very small, have to be quite young to be purely crystalline, and therefore a temperature enhancement either must exist or have existed very recently has existed in order to produce them, thus supporting the idea of an active Dione.

## 4. Conclusions

Dione has been the target of numerous analyses aimed at determining how similar this satellite is to its counterpart Enceladus. Results so far have been contradictory, leaving open the question whether Dione is—or until fairly recently was—an active world. To contribute to this quest, here we examine the wispy terrain on Dione, which consists of bright tectonic faults that crisscross the trailing hemisphere. Our analysis of one of the faults focused on the H_2_O ice phase, specifically the amorphous ice fraction, as it has been shown that variations in temperature will cause a change in the ice phase from amorphous to crystalline (e.g., Mastrapa & Brown 2006).

We apply a technique previously adopted in a similar study of craters on Rhea (Dalle Ore et al. 2015), this time applied to a global mosaic of the surface of Dione and then to a small region where high-spatial-resolution hyperspectral data were available. The resulting fraction of amorphous to crystalline ice was mapped and compared to Dione’s geological features. On the leading hemisphere, the fraction of amorphous ice is less than 10%, consistent with the infalling of E-ring particles known to be composed predominantly of H_2_O in crystalline form. On the trailing hemisphere, there is evidence of amorphous ice present in amounts that vary up to about 30%, corresponding to the darker terrains where magnetosphere material accumulates. This pattern is interrupted by areas that are mostly (and sometimes purely) crystalline. We find a clear correspondence of the pure crystalline regions and the wispy terrain.

We study in detail one of the faults in the wispy terrain, making use of a high-resolution mosaic of the region. Here, the fault is seen to be associated with areas dominated completely by crystalline H_2_O ice, marking those regions that have had temperature enhancements. Based on previous work by Howett et al. (2018), we deduce that the temperature enhancements have remained undetected due to the limited expanse of the area and their relatively low temperature, which we estimate to be in the range between 100 and 120 K.

Based on the fact that the fraction of amorphous to crystalline H_2_O ice in one of the faults is zero or close to zero, and subject to the assumptions and caveats described in Section 3, we estimate an upper limit for the formation age of this region in the Wispy Terrain shown in the high-resolution map. We calculate the timing of the transition from crystalline to amorphous ice due to ion bombardment for a fraction of 5% amorphous H_2_O ice. This amount is significantly higher than the measured fraction in the fault. We obtain a value of <200 Ma for the slightly amorphized ice, implying that the fault has to be even younger. This estimate is lower than the age estimates based on stratigraphic relations (Hirata 2016). This implies that the fault is or has recently been producing crystalline ice, an indication that the enhancement in temperature, although low, is still present at this time, and supporting the idea that Dione is indeed an active world.

## Figures and Tables

**Table 1:** Approximate Ages Corresponding to Varying Amorphous H_2_O Fractions

Amt (%)	0 ± 1	5 ± 1	10 ± 1	15 ± 1	20 ± 1	25 ± 1	30 ± 1
Age (Ma)	0 ± 30	152 ± 31	312 ± 33	482 ± 35	662 ± 37	853–40 + 39	1058–43 + 42

**Table 2:** Ages Corresponding to the Amorphous H_2_O Fraction in the Map

Amt (%)	0 ± 2	6 ± 1	11 ± 1	14 ± 1	16 ± 1	18 ± 1	19 ± 1	20 ± 1	22 ± 1	25 ± 1	27 ± 1	31 ± 2
Age (Ma)	0–60 + 59	183–32 + 31	346–34 + 33	447–35 + 34	517–36 + 35	589 ± 36	625–37 + 36	662 ± 37	737 ± 38	853–40 + 39	933–41 + 40	1100 ± 43

**Figure A1.**
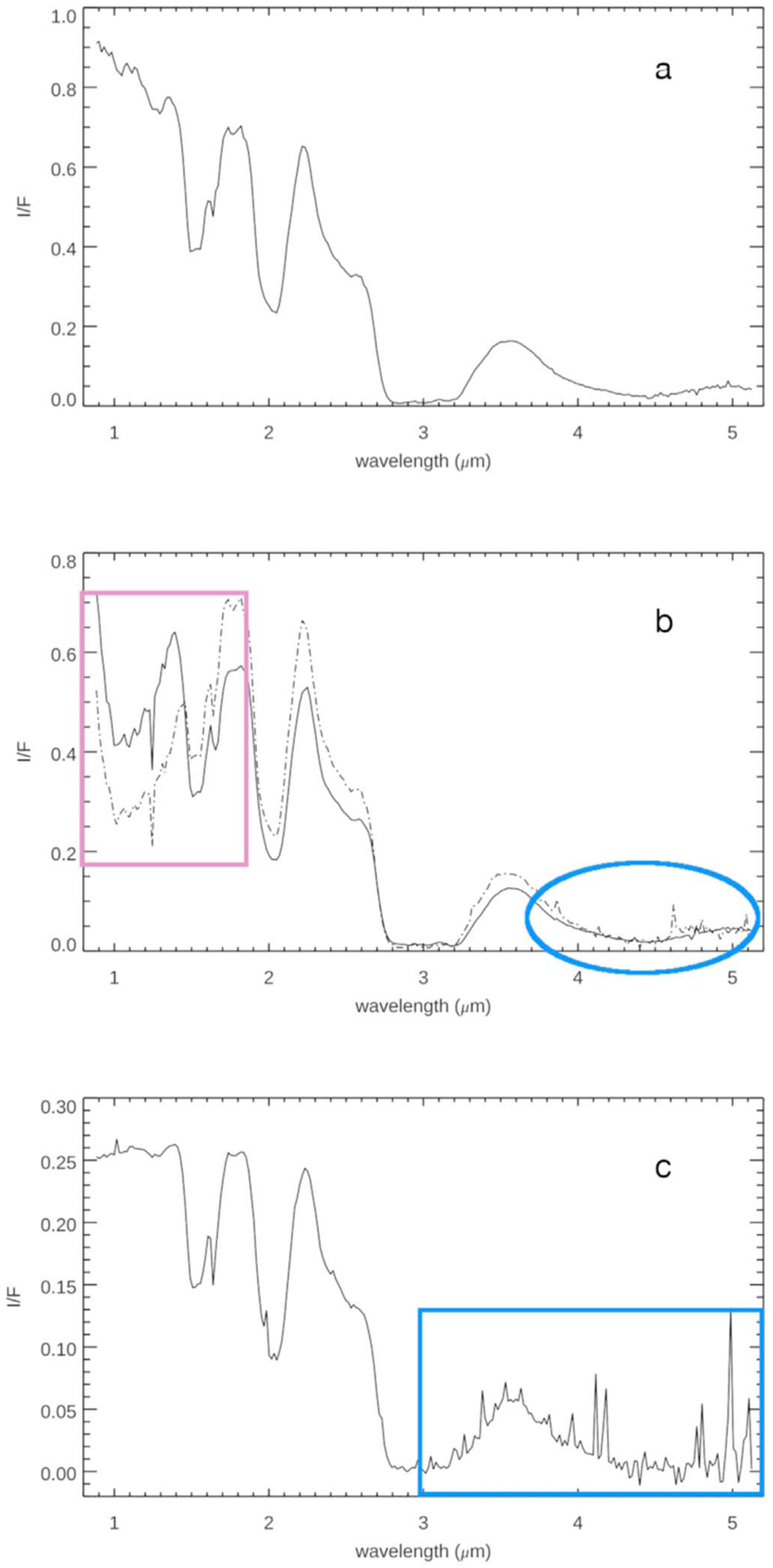
Examples of spectra returned by Cassini VIMS in the 0.9–5.1 *μ*m range. (a) Enceladus’ spectrum with good signal-to-noise ratio (S/N). (b) Enceladus’ spectra showing saturation at short wavelengths. (c) Mimas’ spectrum with low S/N at longer wavelengths.

**Figure A3.**
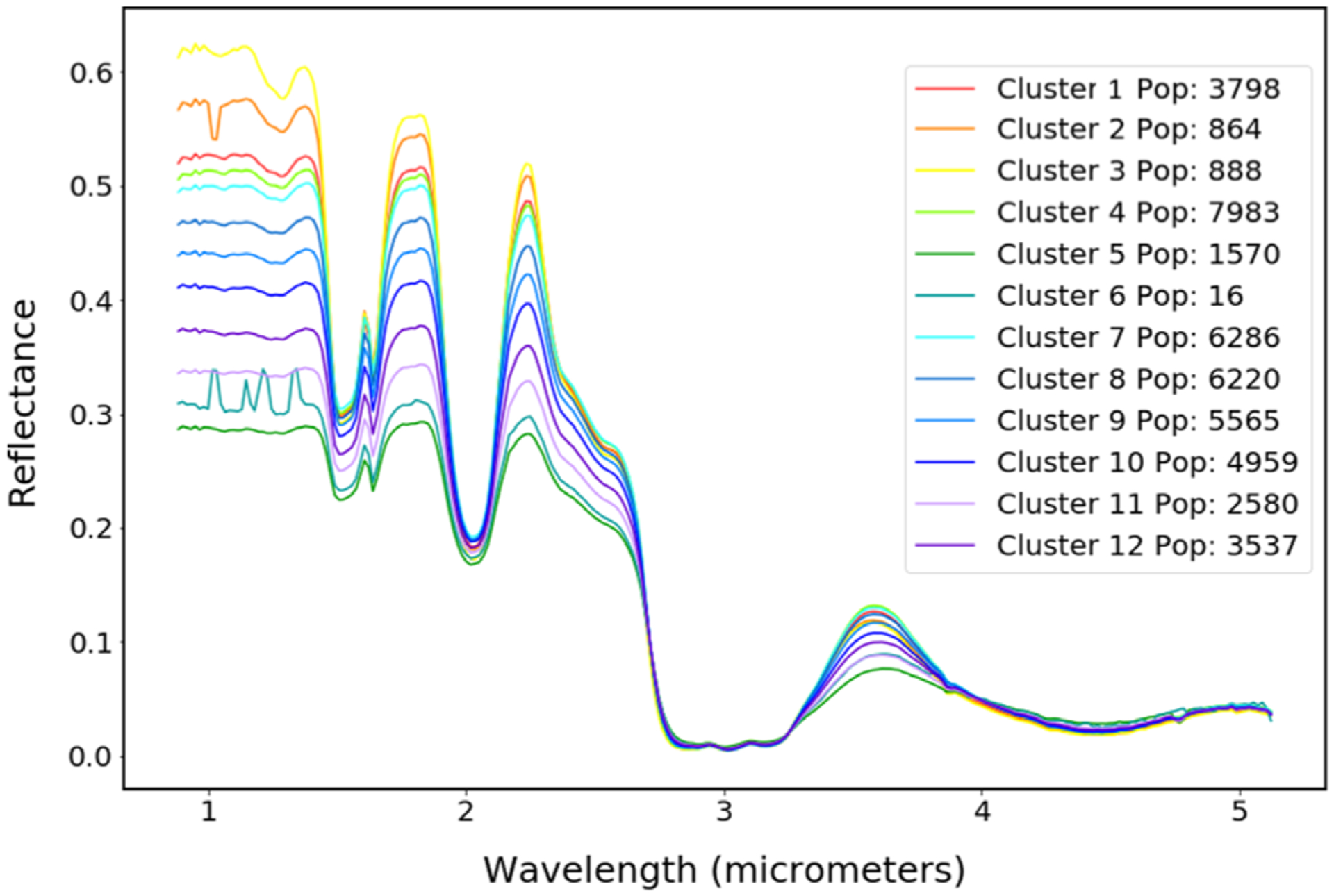
Spectral averages for the pixels included in each of the clusters listed in the legend with their respective population.

**Figure A4.**
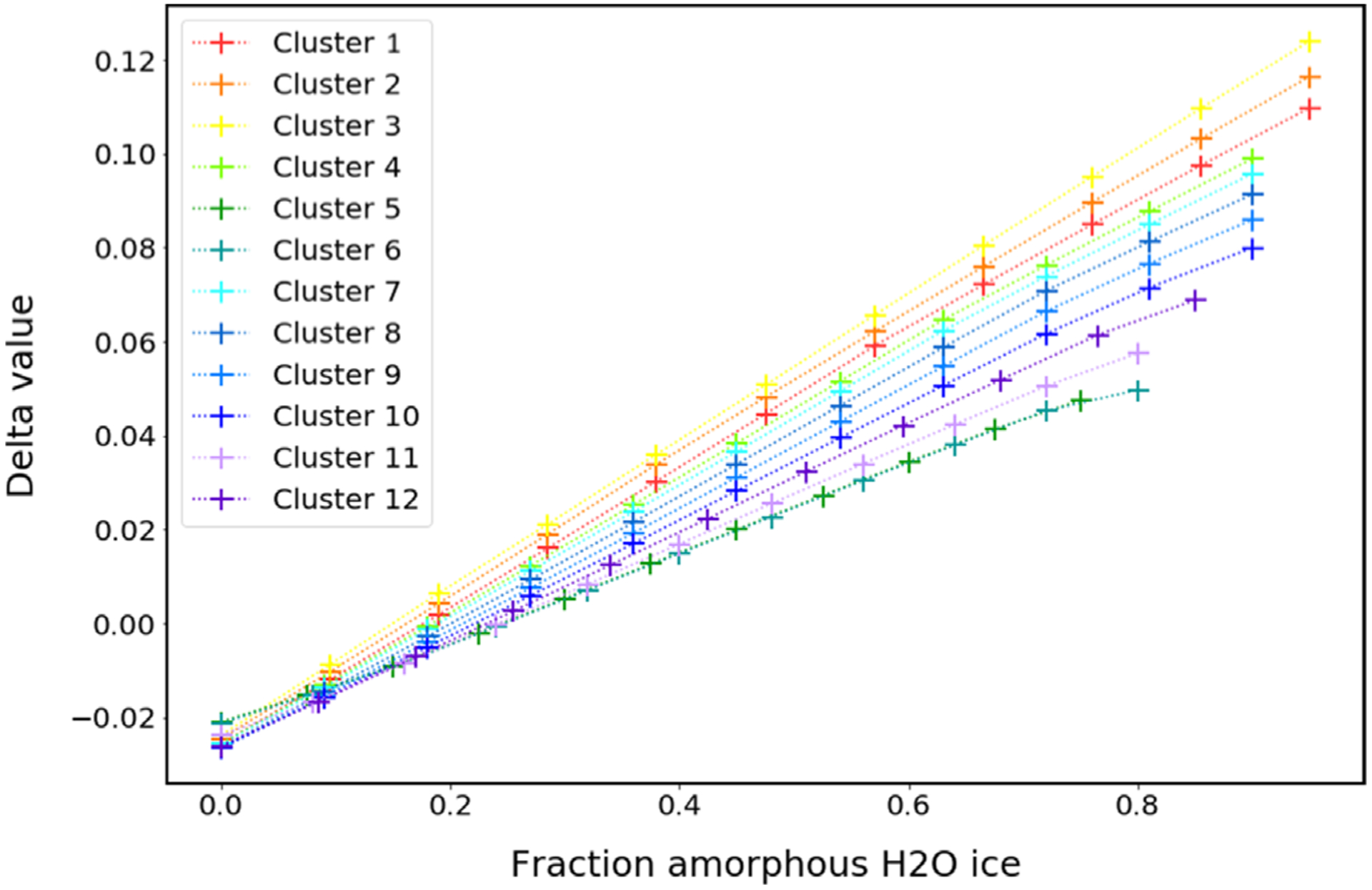
Calibration curves for the spectrally distinct regions of Dione’s surface.

## Footnotes

7 Throughout this paper, we refer to the amorphous to crystalline fraction as equivalent to one minus the crystallinity fraction. Therefore, terms such as crystallinity, amorphous to crystalline, or crystalline to amorphous are inherently equivalent from a physical standpoint.

## Appendix

### A.1. Data Description

For our study, we focused on the infrared channel only. We selected 637 VIMS cubes, acquired by Cassini between 2004 (Sequence S06) and 2012 (Sequence S73). The phase angle ranges between 10° and 50°, while the spatial resolution is in the interval 0.94–241.6 km pixel^−1^.

The data were calibrated using the latest VIMS radiometric calibration, termed RC19 (Clark et al. 2018).

For IR spectra acquired with long exposure times and shorter wavelengths (0.8 up to 3.0 *μ*m) were affected by saturation as shown in Figure A1: panel (a) shows an example of a spectrum not affected by saturation (Enceladus’ spectrum acquired with an 80 ms exposure time); in panel (b), the solid-line spectrum (160 ms exposure) has a less pronounced saturation issue and better signal-to-noise ratio (S/N) at longer wavelengths than the dashed-lined spectrum (260 ms exposure); panel (c) shows a spectrum (Mimas’ spectrum, exposure of 120 ms) with an S/N that is very good at short wavelengths but not as good at longer wavelengths. It is apparent that saturation depends on the exposure time, but the ideal exposure time changes for each satellite. The saturation looks like an absorption band. It starts at short wavelengths (around 0.8 *μ*m), and it can extend up to ~3.0 *μ*m if the flux of photons onto the detector is sufficiently large: the longer the exposure, the deeper and wider the saturation tends to be.

On the other hand, for short integration times, the spectrum longward of 3 *μ*m tends to exhibit poor S/N due to the lower reflectivity of water ice at these wavelengths. Therefore, for certain observations, long exposure times were applied to increase the signal strength at the longest wavelengths, beyond 3.0 *μ*m, even though this meant having to “sacrifice” the shorter wavelengths due to saturation effects. VIMS spectra can also show random spikes, i.e., random artifacts in the spectrum, mainly due to *γ*-rays emitted by the Cassini’s RTG (radio-isotope thermoelectric generator), or to a lesser extent, by energetic particles (e.g., cosmic rays, magnetospheric articles) impacting the detector.

The latest version of VIMSCAL, a routine of the USGS Integrated Software for Imagers and Spectrometers (ISIS), automatically removes the saturation effect by deleting the spectral channels affected by this issue.

To account for the noise affecting the long-wavelength portion of the spectrum, we rejected data from channels that exhibit low S/N, estimated by following the method described in Stoehr et al. (2008). The signal is calculated as the median of the flux at each wavelength *i*, and the noise as:

(2)
$$\text{noise=}\frac{1.482602}{\sqrt{6}*\text{median}\left(\left|\left(2*\text{flux}\left(i\right)-\text{flux}\left(i-2\right)-\text{flux}\left(i+2\right)\right)\right|\right)}.$$

We applied Equation (2) to all VIMS spectra in the range 2.9–5.1 *μ*m to calculate the S/N, and we set a threshold level of S/N = 10 in order to discriminate between good (S/N > 10) and bad (S/N < 10) spectra. With this technique, we were able to keep almost all the original selected data sets. Indeed, even if a portion of the spectrum was discarded because of saturation or noise, there was no reason to reject the whole spectrum, or more generally, to exclude a VIMS cube because a portion of it had saturation/noise issues, thus maximizing the scientific return of Cassini/VIMS.

VIMS cubes were also photometrically corrected. In fact, the combination of spectra from different observations shows photometric effects due to the variability of illumination and geometry conditions. Our photometric correction first accounts for the effects of the large-scale geometry of illumination and observation, due to the curvature of Dione, by applying the Akimov disk-function (1975, 1980, 1988; Shkuratov et al. 1999a). To further correct the effects induced by photometry, we first plotted, for each VIMS-IR channel, the I/F versus the phase angle of each pixel of the whole data set, and then we fit the data with a third-order polynomial function.

Finally, VIMS data were cylindrically projected on a common surface and combined in a hyperspectral mosaic, sampled at a fixed resolution of 1°lat × 1°lon. Geometry calculations have been performed for every VIMS pixel by using the SPICE kernels and libraries provided by NASA’s Navigation and Ancillary Information Facility (Acton 1996).

### A.2. Note on Data Analysis

We should point out that another parameter, the presence of nano-sized grains on the surface, could affect the measurement of the ice fraction affecting, as shown in Clark et al. (2012), the shape of the 2.0 *μ*m band. However, when looking at Clark et al. (2012) Figure 22(a) it is evident that the presence of nano-sized grains pushes the bottom of the 2.0 *μ*m band toward longer wavelengths, which practically would correspond to a “super crystalline” case. Indeed, when mixed with amorphous ice, the bottom of the 2.0 *μ*m band shifts to shorter wavelengths and does not encroach into the nanograin scenario.

#### A.3. Phase Fraction Measurements on Dione

##### A.3.1. Calibration

To achieve this goal, we identified regions that were spectrally distinct on Dione’s surface as determined by applying a cluster tool to the 1.5 *μ*m band (Figure A2). We chose this spectral region to identify areas with distinct spectral characteristics related to grain size, composition, and temperature. The adopted clustering code is based on a K-means classification approach (Marzo et al. 2006, 2008, 2009) and implemented with the Calinski & Harabasz (1974) (CH) criterion, which allows the identification of the best number of clusters representing the data set. The clustering was applied twice. The first time was to identify noisy pixels to be removed. The second clustering, limited to the less noisy pixels, yielded 13 as the best number of classes describing the spectral variations around the 1.5 *μ*m band (from ~1.2 to 1.6 *μ*m). For each cluster, we calculated the average of all pixels belonging to it, as shown in Figure A3.

For each cluster average, we calculated a best-fitting model by making use of the scattering radiative transfer Shkuratov approach (Shkuratov et al. 1999b). From the model, we built a grid of synthetic spectra and varied in locksteps the phase of the H_2_O ice, going from zero to fully amorphous in intervals of approximately 10%.

The models included H_2_O ice and amorphous carbon (AC) in order to account for the darkening and therefore the lower albedo of some of the regions. In the initial best-fit models, H_2_O ice was assumed to be fully crystalline. After experimenting with three different H_2_O ice temperatures (80, 100, and 120 K), it was determined that 100 K yielded the best model fits to the cluster averages, even though the difference among temperatures was not always significant. Although the surface temperature changes across the surface, depending on exposure to the Sun, the difference is not sufficient to produce a significant impact on the models. The optical constants adopted in the modeling were those of Mastrapa et al. (2008) for the H_2_O ice and of Rouleau & Martin (1991) for the amorphous carbon. We focused our modeling effort on matching as closely as possible the region around the 2 *μ*m band, which is where our analysis is centered. The relative amounts and grain sizes obtained for the models are listed in Table A1 for both global and high-resolution mosaics. For the high-resolution case, the slightly higher-temperature fits were favored with the exception of clusters 4, 8, 9, and 11. Since these clusters are not distinct in any obvious way from the others, the difference in temperature probably indicates that we have reached the limit of what we can deduce from the data and the difference in temperature is not significant.

The calibration curves for each cluster were calculated by building a grid of models, starting with the best-fit model and changing the relative amount of amorphous to crystalline H_2_O ice in locksteps of ~10%. The grain size of amorphous and crystalline H_2_O was assumed to be the same. For every synthetic spectrum, we measured the value of the “delta” parameter. Ultimately, for each cluster, we obtained a calibration curve as shown in Figure A4.

##### A.3.2. Delta Measurement

We named the change in the 2.0 *μ*m band shape “delta.” In essence, the technique hinges on the fact that the 1.5 and 2.0 *μ*m bands can be fitted with Gaussians with different width and position (Grundy et al. 1998). While the 1.5 *μ*m band is a combination of several Gaussians, the 2.0 *μ*m band is composed of three Gaussians, of which one is predominant in its contribution to the total absorption (see Figure 3 in Grundy et al. 1998). Variations in ice phase affect the relative strength of the subsidiary Gaussians that compose the 2.0 *μ*m band (Mastrapa et al. 2008).

**Table A1:** Parameters for the Best-fitting Models of the Cluster Averages in the Global (Left Columns) and High-resolution (Right Columns) Maps

Cluster	GLOBAL MAP, T 100K				HI-RES MAPT 100 K				HI-RES MAP T 120 K			
	H_2_O fraction	H_2_O grain size (*μ*m)	AC fraction	AC grain size (*μ*m)	H_2_O fraction	H_2_O grain size (*μ*m)	AC fraction (*μ*m)	AC grain size (*μ*m)	H_2_O fraction	H_2_O grain size (*μ*m)	AC fraction	AC grain size (*μ*m)
1	0.95	15.75	0.05	20.00	0.88	9.00	0.12	20	0.875	9.00	0.125	20
2	0.96	16.50	0.04	20.00	0.84	9.00	0.16	20	0.84	9.00	0.16	20
3	0.96	17.75	0.04	20.00	0.82	9.00	0.18	20	0.82	9.20	0.18	20
4	0.95	15.00	0.05	20.00	0.91	9.15	0.09	20	0.90	9.20	0.10	20
5	0.81	11.50	0.19	20.00	0.865	9.35	0.135	20	0.865	9.70	0.135	20
6	0.83	11.25	0.17	20.00	0.92	9.35	0.08	20	0.92	9.50	0.08	20
7	0.94	14.50	0.06	20.00	0.84	9.50	0.16	20	0.84	9.50	0.16	20
8	0.93	14.25	0.07	20.00	0.88	9.60	0.12	20	0.88	10.00	0.12	20
9	0.92	13.75	0.08	20.00	0.88	9.95	0.12	20	0.88	10.20	0.12	20
10	0.90	13.25	0.10	20.00	0.928	10.25	0.072	20	0.925	10.20	0.075	20
11	0.86	12.00	0.14	20.00	0.90	10.65	0.095	20	0.90	11.00	0.10	20
12	0.88	12.75	0.12	20.00	0.92	11.25	0.08	20	0.92	11.50	0.08	20

**Note.** Values in parentheses are grain sizes.

As shown in detail in Dalle Ore et al. (2015), the delta measurement is obtained by comparing the shape of the 2.0 *μ*m band with a Gaussian function that is supposed to represent the “core” of the band. The residual signal has two minima at 1.95 *μ*m and 2.1 *μ*m, and their relative reflectance yields delta.

##### A.3.3. Phase Fraction Determination

The last step in determining the fraction of amorphous to crystalline H_2_O ice is to measure the delta parameter for every pixel on the surface and then associate its value, through the correct calibration curve, to the fraction of amorphous ice. The resulting phase fraction map is shown in Figure 1, where the phase map is overlaid upon the Dione base map (Schenk et al. 2011).
